# Assessment of open surgery suturing skill: Simulator platform, force-based, and motion-based metrics

**DOI:** 10.3389/fmed.2022.897219

**Published:** 2022-08-30

**Authors:** Irfan Kil, John F. Eidt, Richard E. Groff, Ravikiran B. Singapogu

**Affiliations:** ^1^Department of Electrical and Computer Engineering, Clemson University, Clemson, SC, United States; ^2^Division of Vascular Surgery, Baylor Scott & White Heart and Vascular Hospital, Dallas, TX, United States; ^3^Department of Bioengineering, Clemson University, Clemson, SC, United States

**Keywords:** medical simulator, objective metrics, sensor informatics, suturing, skill assessment

## Abstract

**Objective:**

This paper focuses on simulator-based assessment of open surgery suturing skill. We introduce a new surgical simulator designed to collect synchronized force, motion, video and touch data during a radial suturing task adapted from the Fundamentals of Vascular Surgery (FVS) skill assessment. The synchronized data is analyzed to extract objective metrics for suturing skill assessment.

**Methods:**

The simulator has a camera positioned underneath the suturing membrane, enabling visual tracking of the needle during suturing. Needle tracking data enables extraction of meaningful metrics related to both the process and the product of the suturing task. To better simulate surgical conditions, the height of the system and the depth of the membrane are both adjustable. Metrics for assessment of suturing skill based on force/torque, motion, and physical contact are presented. Experimental data are presented from a study comparing attending surgeons and surgery residents.

**Results:**

Analysis shows force metrics (absolute maximum force/torque in z-direction), motion metrics (yaw, pitch, roll), physical contact metric, and image-enabled force metrics (orthogonal and tangential forces) are found to be statistically significant in differentiating suturing skill between attendings and residents.

**Conclusion and significance:**

The results suggest that this simulator and accompanying metrics could serve as a useful tool for assessing and teaching open surgery suturing skill.

## 1. Introduction

Objective measures of surgical skill have remained elusive because of a lack of consensus regarding the optimal metrics. Surgical trainees are often evaluated by surgical educators using subjective rating scales that often lack precision and reproducibility. Precise quantification of metrics that define “best surgical practices” factors would have potential value to certifying organizations, credentialing committees and surgical educators in addition to providing surgeons in training with objective feedback. Quantification of a surgeon's skill has received attention in recent years due to multiple factors including: duty hour restrictions on surgical residents, limited training opportunities, a call for the reduction in medical errors, and a need for structured training (1–4). Surgical skill is important due to the direct relationship between surgical performance and clinical outcomes such as hospital readmission and complication rates (5). Surgical outcomes may be improved through training to improve skill. For this purpose, surgical simulators —capable of simulating an aspect of a surgical procedure and of assessing and/or training the subject's skill on a given task— have received special attention in recent years. One main advantage of using simulators is the ability to train surgical skills without the use of humans or animals. Another key advantage is the ability to measure skill and its progression over time. Overall, evidence suggests that surgical simulators are potentially effective in training surgical skills (6, 7).

A variety of surgical simulators have been developed to aid surgeons-in-training in the acquisition of a wide range of surgical skills. Surgical simulators may be as basic as simple devices that allow surgeons to practice suturing of synthetic materials (e.g., sponges, plastic tubes) to highly sophisticated computer-based virtual operating rooms. The most effective currently available simulators focus on minimally invasive techniques used in endovascular, laparoscopic, and robotic procedures. Simulation of open surgical techniques has historically relied on the use of animal or cadaver labs which frequently lack object performance metrics (8–12). Suffice it to say that there is a need for more precise, objective measures of open surgical skill (13).

Traditional surgical training shares many features in common with other apprenticeship-based skilled trades. Promotion is often based on duration of service rather than objective demonstration of specific objective performance metrics. This type of training may be highly subjective since feedback often depends on the expert surgeon's preferences and style. Further, training draws expert surgeons away from clinical responsibilities (14). Simulators were developed to address these problems and to standardize and automate assessment of a surgeon's skill. Researchers have focused on identifying objective metrics for surgical skill and on developing devices to measure these metrics. Objective metrics establish a basis for continued practice in order to achieve defined performance goals.

Many metrics for skill assessment have been presented in the literature. These metrics can be classified as force-based metrics (15–23), motion-based metrics (8, 18, 23–30) and image-based metrics (29, 31–39). Force-based metrics, such as absolute, mean, and peak forces and force volume (16–18, 23), have been most successful at distinguishing novice vs. expert performance at surgical tasks. Hand and/or surgical tool motion obtained *via* sensor-based kinematic data were also examined to extract motion-based metrics, which can distinguish skill level (8, 18, 28–30). Acceleration of the hand and rotation of the wrist were found to distinguish expert surgeons from novices (18, 28). In addition, hand and/or surgical tool motion obtained from external video using Artificial Intelligence (AI) were also examined to extract motion-based metrics (36–39). Total duration, path length, and number of movements were found to be important for distinguishing between attendings and medical students (37). Further, computer vision has also been used to extract image-based metrics as a means to quantify surgical skill (31, 32, 34, 35). Frischknecht et al. (31) analyzed photographs taken post-procedure to assess suturing performance. Metrics that proved most meaningful in ranking the quality of suturing included the number of stitches, stitch length, total bite size, and stitch orientation.

Suturing is a fundamental surgical skill required in a variety of operations, ranging from wound repair in trauma care to delicate vascular reconstruction in vascular surgery (40). The process of suturing can be divided into the following phases: (i) puncturing a needle into the tissue perpendicularly, (ii) driving the needle through the tissue following the curvature of the needle, (iii) exiting the tissue from an exit point, and (iv) withdrawing the needle from the tissue completely prior to tightening the suture. Learning skilled suturing is essential for novice medical practitioners and has been incorporated into most fundamental skills training curricula, for example, the Fundamentals of Laparoscopic Surgery (FLS) (6, 41, 42) and Fundamentals of Vascular Surgery (FVS) curricula (43). However, most currently available simulators for teaching suturing have been developed for minimally invasive surgery (44); only a handful of attempts have focused on open surgery (45–47). Furthermore, the majority of studies that examine suturing skill focus on product metrics, i.e., metrics based on analyzing the final results of the task. Process metrics, i.e., metrics that quantify skill by analyzing how the task was performed, provide significantly more insight for skill training and assessment than product metrics but are also more technically challenging to obtain.

To address the limitations of current surgical simulators, we have developed a suturing simulator which collects synchronized force, motion, touch, and video data as trainees perform a prespecified suturing task. Product and process metrics are extracted from these data and are used to distinguish suturing skill level. A feature of this system is that standard surgical tools (needle holder, needle with surgical thread, etc.) are used on the platform in contrast to simulators which require the use of modified surgical tools (for example needle coloring, dots for computer vision tracking, etc.). Inspired by suggestions from collaborators in vascular surgery, the system simulates suturing at various depth levels, which represent surgery inside a body cavity or at the surface. Suturing at depth is especially important in vascular surgery and requires significantly different and less intuitive hand motions as compared to suturing at the surface (48).

The suturing simulator presented here extends a preliminary version of the platform presented in Kavathekar et al. (49) and Singapogu et al. (50) that featured a single external camera, a force sensor, and a motion sensor. This paper presents the construction of the simulator, metrics based on force, motion and touch, and a study of attending and resident surgeons toward skill assessment using these metrics. The study was carried out with three main objectives: (1) to validate the simulator's capability of collecting synchronized force, motion, touch, and video data, (2) to extract metrics from data collected from a population with open surgery suturing experience, (3) to test the construct validity of the various metrics. The paper is organized as follows. Section 2 describes the simulator, experimental setup, and methods used in the study. Section 3 presents the experimental results, along with a discussion of the force- and motion-based metrics. Section 4 presents conclusions and future work.

## 2. Materials and methods

### 2.1. The suturing simulator

#### 2.1.1. Simulator platform

**Figure 1:**
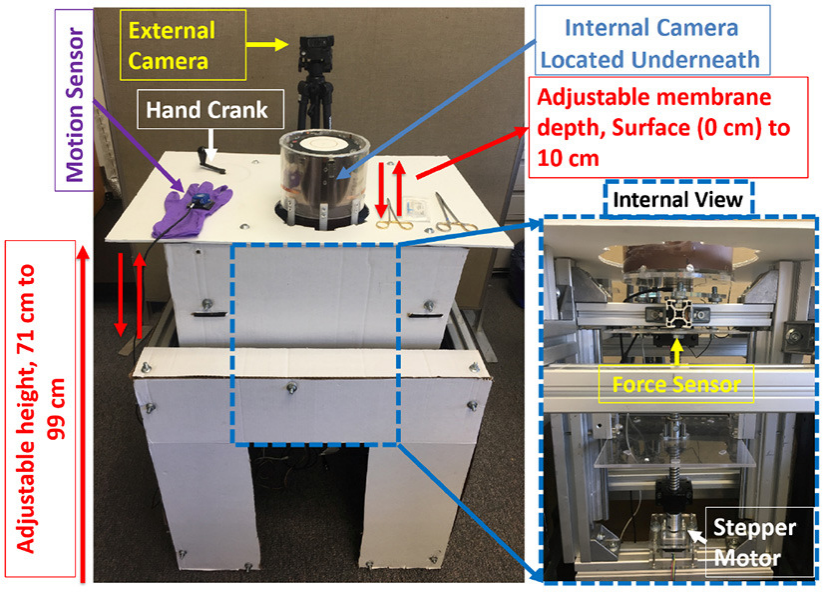
Suturing simulator overview.

**Figure 2:**
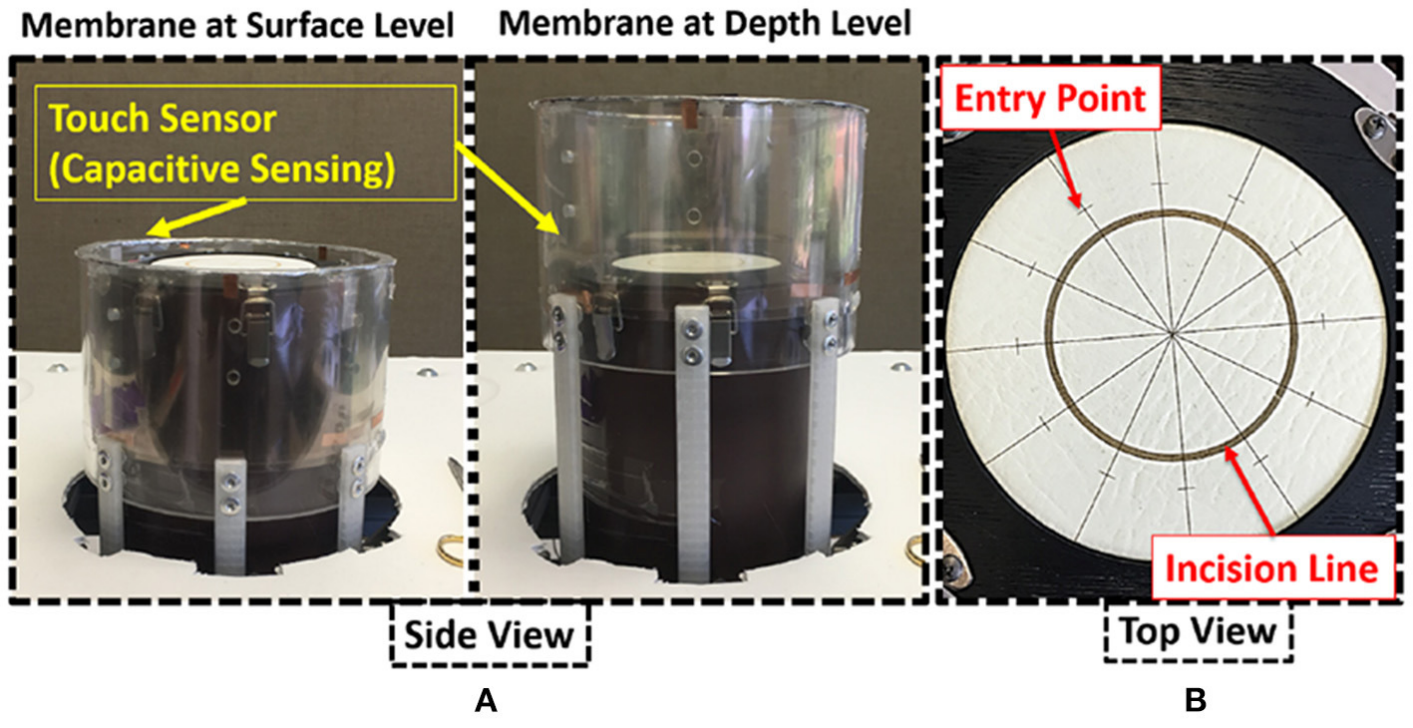
**(A)** membrane housing, **(B)** suture membrane.

The physical system was designed with the following main components: (a) membrane housing, and (b) height adjustable table (see Figure 1). The cylindrical membrane housing was constructed from clear acrylic and its sides were shielded externally with an aluminum sheet. Eight metal latches along the upper exterior of the membrane housing were used to secure the membrane, a material such as GoreTex®, artificial leather, or other fabric, on which suturing is performed (see Figure 2A).

Similar to the radial suturing task in the Fundamentals of Vascular Surgery (43, 51), the suture membrane (see Figure 2B) was designed such that suturing is performed in a radial and uninterrupted fashion. A circle, representing an incision, was drawn on the membrane. The circle was partitioned by radial lines into equal sections each spanning 30°, similar to a clock face. Needle entry points were marked on the radial lines. The distance of the entry mark from the incision line is based on the diameter of the needle. The marks indicated where suturing was to be performed (entry on one side, exit on the other). All membranes were made of artificial leather using a laser cutter.

An internal camera (Firefly MV USB 2.0, Point Grey Research Inc., British Columbia, Canada) was positioned inside the membrane holder and used to record needle and thread movement from underneath the membrane. White LED strips were mounted inside the membrane housing to provide consistent lighting conditions. In addition, an external camera (C920 HD USB 2.0, Logitech International S.A., Lausanne, Switzerland) was positioned above the membrane to record the membrane and hand movement of the subjects during suturing.

**Figure 3:**
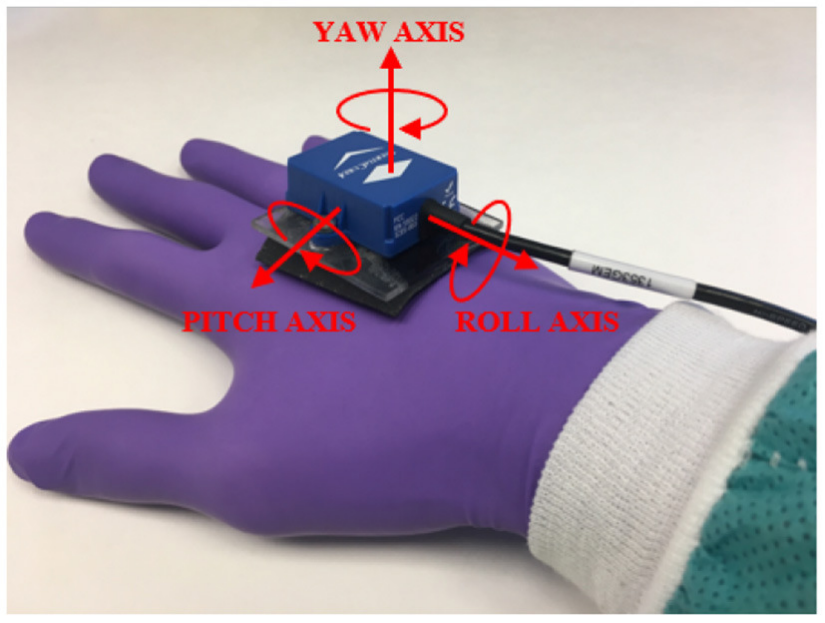
Motion data collected using InertiaCube4 sensor placed on dorsum of subject's hand.

A 6-axis force/torque sensor (ATI MINI 40, ATI Industrial Automation Inc., NC, USA) was placed under the housing to measure forces and torques applied to the membrane during suturing (see Figure 1). An InertiaCube4 sensor (InterSense Inc., MA, USA) was used to record hand motion during suturing (see Figure 3).

To simulate suturing in a body cavity or at the surface of the body, a transparent acrylic cylinder is positioned around the membrane holder. The vertical position of the cylinder can be adjusted to simulate suturing at different depths (see Figure 2A).

Capacitive sensing was employed to detect physical contact, i.e., touch, between the subjects' body or the surgical instrument and the cylinder. The interior and top of the cylinder was lined with flexible conductive film (Indium Tin Oxide coated plastic sheet) and aluminum foil, respectively. The conductive materials were attached to a simple capacitive sensing circuit and read using an Arduino.

The membrane housing was mounted onto an adjustable height table. This allows subjects to set the height of the platform as desired for comfort during the suturing exercise (48). Ergonomic studies of the height of operating tables show that the optimum height of the table lies between 55 cm and 100 cm from the floor up to table surface (52–54). The table for the suturing simulator was modified to permit heights between 71 cm and 99 cm.

**Figure 4:**
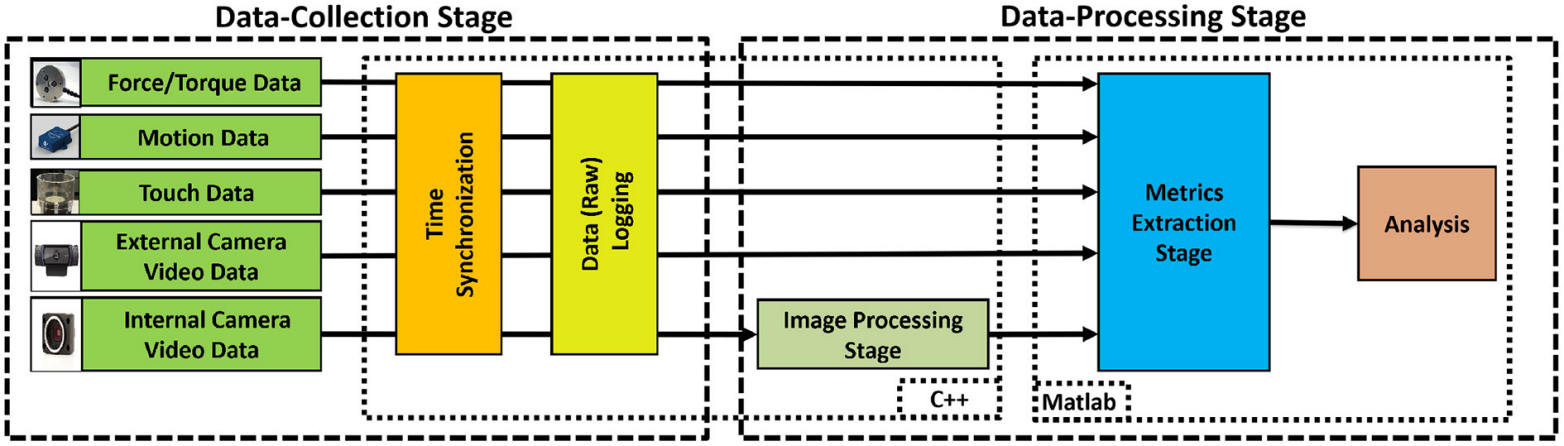
System process flow-chart, consisting of two stages. In the Data Collection Stage, raw data from multiple sensors were synchronized and logged. In the Data Processing Stage, the collected data were used to extract metrics for suturing skill.

The system processes of the suturing simulator (see Figure 1) are categorized into two main stages: (i) Data Collection, and (ii) Data Processing (Figure 4). In the Data-Collection stage, the system synchronizes and logs force, motion, video, and touch data during suturing. The Data-Processing stage uses the collected data to extract metrics of suturing skill.

#### 2.1.2. Data-collection stage

Data were collected from the four sensing modes: force/torque, motion, video, and physical contact. Force/torque data were collected using the 6-axis force/torque sensor and logged at 1 kHz during suturing. To obtain force/torque data from the sensor, software was written using the NI-DAQ Software Development Kit (SDK). Collected force/torque data were filtered offline with a 10^th^-order Butterworth lowpass zero-phase filter with a cutoff frequency of 50 Hz to remove noise and smooth the data. To record hand motion, the InertiaCube4 sensor was placed on the dorsum of the subject's dominant hand as shown in Figure 3 and logged at 200 Hz during suturing. InterSense SDK was used to obtain θ_*yaw*_, θ_*pitch*_, and θ_*roll*_ measurements of the subject's wrist motion. The internal camera with FlyCapture SDK was used to record needle and suture motion from under the membrane at 60 fps. The external camera was used to record membrane and hand movement at 30 fps. An open source computer vision library (OpenCV 3.0.0) was used to capture and log the external video. For logging touch data, the Arduino capacitive sensing (55) and serial communication libraries (56) were used.

We modified the previous system to include an internal camera, enabling extraction of vision-based metrics. In the previous system, synchronization of the data stream was achieved in post-processing, whereas our current platform synchronizes data collection on a single PC using a multithreaded implementation and timestamping. The Data Collection Stage software was written in C++ using Microsoft Visual Studio 2013 (57).

During suturing, all unprocessed (raw) data is synchronized and logged. Logging allows for revisiting the raw data at any time for additional investigation and analysis. The raw data were then used in the Data-Processing stage.

#### 2.1.3. Data-processing stage

In this stage (see Figure 4), internal video was first processed with a computer vision algorithm to obtain information about needle and thread movement (33). This information was then used to identify the individual suture cycles. Next, collected raw data were used to extract metrics for each time the subject is actively suturing.

##### 2.1.3.1. Vision-enabled partitioning of suture cycle

During continuous suturing, a single suture cycle can be divided into two distinct periods of time: active suturing time and idle time. Active suturing time is the time between needle entry into the membrane and complete needle removal from the membrane. Idle time is the time between the end of one active suturing time to the start of the next. In other words, active suturing is the time taken by subjects to complete one suture, whereas idle time is the time spent preparing for the next suture. Active suturing time may be further decomposed into 4 phases: a) entry phase—puncturing the needle into the tissue; b) driving phase—driving the needle along some path under the membrane; c) exit phase—exiting the needle tip from the tissue; and d) pull-out phase—pulling the needle completely from the tissue and then tightening the thread.

**Figure 5:**
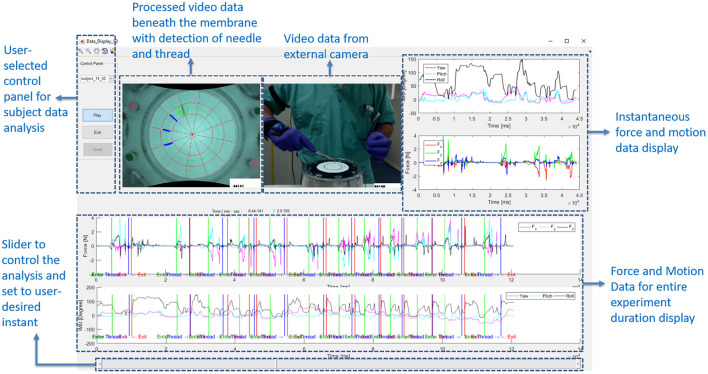
Graphical User Interface (GUI) designed to show the synchronized force and motion data, along with videos from external and internal cameras. GUI allows for convenient, interactive investigation of synchronized data.

**Figure 6:**
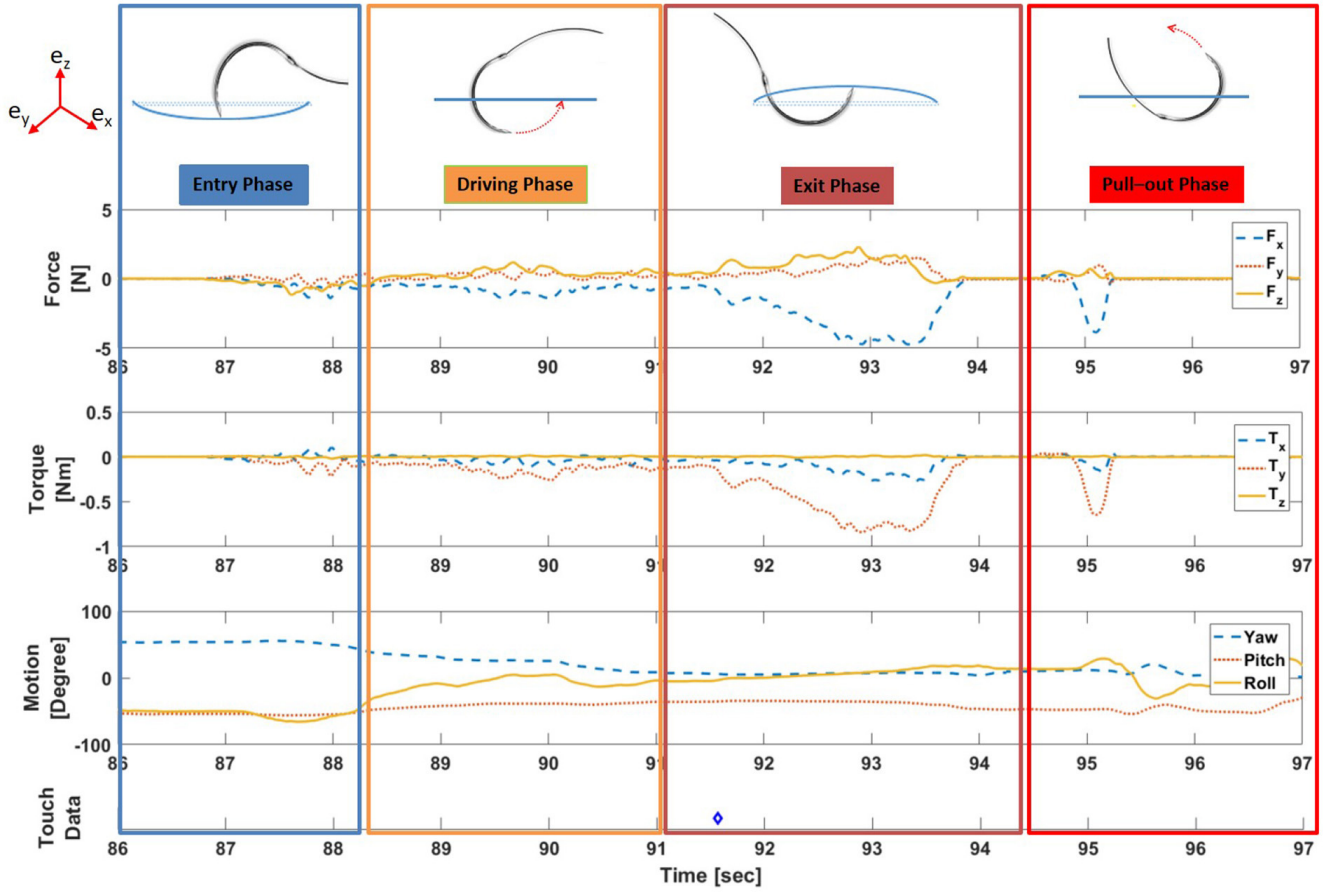
Example of synchronized force, torque, motion, and touch data for one active suturing time with suture sub-events labeled. (Note: Blue diamond symbol (◇) in touch data indicates the time instance of the physical touch).

Dividing each suture cycle into distinct phases allows for context-specific interpretation of the sensor data. Needle entry and exit times obtained from the computer vision algorithm were used to extract each suture cycle for individual analysis. In addition, a Graphical User Interface (GUI) in MATLAB (Figure 5) was created to display synchronized force, motion, and touch data, as well as video from external and internal cameras. The interface also labels the needle entry, needle exit and thread entry times automatically determined by computer vision. The interface enables convenient, interactive exploration of the synchronized data. An example of synchronized data for one active suturing time with the suture sub-events identified (entry, driving, exit, and pull-out phase) is shown in Figure 6.

#### 2.1.4. Metrics for skill assessment

Many of the metrics presented in this paper are computed from time series data of a scalar signal *X*(*t*) using one of the following functions:


(1) $$\begin{array}{r} {\text{PEAK}}_{+}\left(X\right)=\underset{t}{\max}\left(X\left(t\right)\right) \end{array}$$

(2) $$\begin{array}{r} {\text{PEAK}}_{-}\left(X\right)=\underset{t}{\max}\left(-X\left(t\right)\right)=-\underset{t}{\min}\left(X\left(t\right)\right) \end{array}$$

(3) $$\begin{array}{r} \text{PP}\left(X\right)={\text{PEAK}}_{+}\left(X\right)+{\text{PEAK}}_{-}\left(X\right) \end{array}$$

(4) $$\begin{array}{r} \text{INTA BS}(X)=\int_{t}|X(t)|dt \end{array}$$

(5) $$\begin{array}{r} \begin{array}{c} \text{DER}(X)=\sqrt{\int_{t}{\left(\frac{dX(t)}{dt}\right)}^{2}dt} \end{array} \end{array}$$

The time interval over which the maximum is taken is specified in the definition of the specific metric. Typically the time interval corresponds to one whole active suture time. Note that PEAK_+_(*X*) is the maximum value that signal *X* took over the time interval and PEAK_*-*_(*X*) is the negative of the minimum value that signal *X* took during the time interval. If signal *X*(*t*) is negative at some point, then PEAK_*-*_(*X*) can be interpreted as the magnitude of peak negative value of *X*(*t*). PP(*X*) is the peak-to-peak amplitude of signal *X*. As in Trejos et al. (15) and Horeman et al. (16), INTABS(*X*) is related to the impulse for a force signal *X*(*t*). This quantity will be high when *X*(*t*) is high in magnitude over a long period of time. DER(*X*) is the derivative of the signal *X*(*t*) calculated similar to Trejos et al. (15) and can be interpreted as the consistency of signal *X*(*t*) during the time interval.

##### 2.1.4.1. Force/torque-based metrics

**Figure 7:**
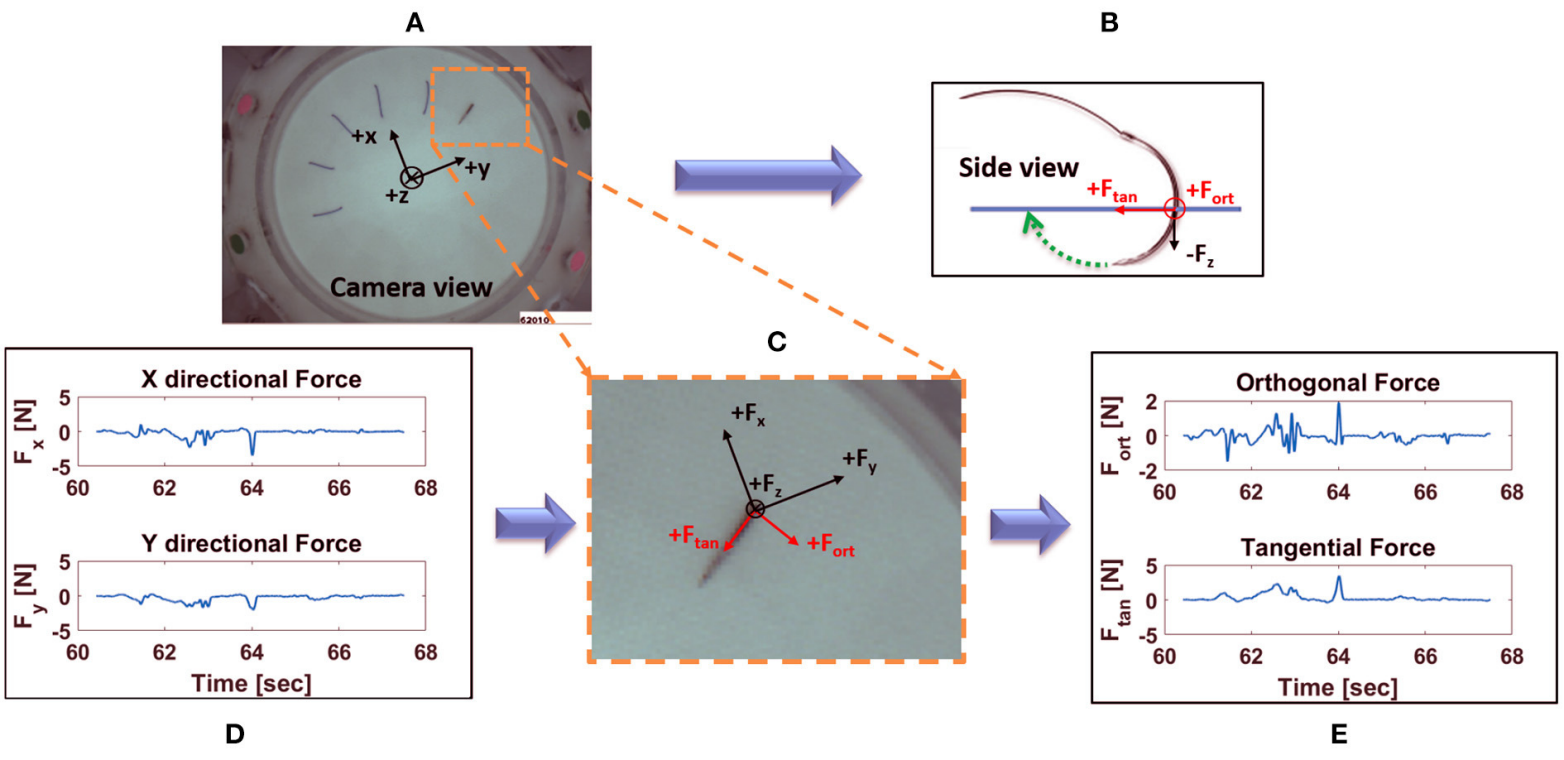
Decomposition of horizontal forces into forces orthogonal and tangential to stitch direction: **(A)** view of the needle taken from internal camera with force sensor coordinate system overlaid; **(B)** side view of the needle along with orthogonal and tangential force direction; **(C)** zoomed in view of the needle, together with the force sensor, orthogonal and tangential forces; **(D)** X and Y directional forces at suture location in **(A)**, for one active suture time; **(E)** corresponding orthogonal and tangential forces from **(A)**, for one active suture time.

For each active suturing time, (1)–(5) were used to compute metrics based on time series for force components *F*_*x*_, *F*_*y*_, and *F*_*z*_, and torque components *T*_*x*_, *T*_*y*_, and *T*_*z*_. Based on the coordinate axes (shown in Figure 7), PEAK_+_(*F*_*z*_) is the maximum force component applied upward on the membrane while PEAK_*-*_(*F*_*z*_) is the maximum force component applied downward (49).

##### 2.1.4.2. Vision-enabled force metrics

Force applied orthogonal to the stitch direction may increase tissue tearing and should therefore be minimized. The axes of the force sensor are not generally aligned with the directions of the radial stitches, so a change of coordinates is required to determine the force components orthogonal and tangential to the stitch direction. Using the suture entry and exit points detected by computer vision (33), the suture direction at each suture location can be identified. Then, a change of coordinates can be applied to compute the force tangential to stitch direction and orthogonal to stitch direction (see Figure 7). Calculations of the orthogonal and tangential forces were achieved as follows.

Total force, $\vec{F}$, can be expressed in the vision coordinate system as:


(6) $$\begin{array}{r} \vec{F}=F_{x}{\vec{e}}_{x}+F_{y}{\vec{e}}_{y} \end{array}$$

where *F*_*x*_ and *F*_*y*_ are the component forces in x and y direction, respectively, as read from the force sensor, and ${\vec{e}}_{x}$ and ${\vec{e}}_{y}$ are the unit vectors in the vision coordinate frame aligned with the x- and y- axes of the force sensor, respectively. Since the coordinate system of the force sensor is constant, ${\vec{e}}_{x}$ and ${\vec{e}}_{y}$ were also constant, independent of suture location. The unit vectors ${\vec{e}}_{x}$ and ${\vec{e}}_{y}$ were precomputed based on a calibration experiment.

The same force can also be represented as


(7) $$\begin{array}{r} \vec{F}=F_{o}{\vec{e}}_{o}+F_{t}{\vec{e}}_{t} \end{array}$$

where *F*_*o*_ and *F*_*t*_ are the component forces orthogonal and tangential to the stitch direction in vision coordinate frame, respectively, and ${\vec{e}}_{o}$ and ${\vec{e}}_{t}$ are the corresponding unit vectors in the vision coordinate frame.

Thus, (6) and (7) can be rearranged as follows to obtain orthogonal and tangential component forces, *F*_*o*_ and *F*_*t*_:


(8) $$\begin{array}{r} \left[\begin{array}{c} F_{o}\\ F_{t} \end{array}\right]={\left[\begin{array}{cc} {\vec{e}}_{o} & {\vec{e}}_{t} \end{array}\right]}^{-1}\left[\begin{array}{cc} {\vec{e}}_{x} & {\vec{e}}_{y} \end{array}\right]\left[\begin{array}{c} F_{x}\\ F_{y} \end{array}\right]. \end{array}$$

Contrary to ${\vec{e}}_{x}$ and ${\vec{e}}_{y}$, the direction of unit vectors ${\vec{e}}_{o}$ and ${\vec{e}}_{t}$ depend on suture location. The vectors ${\vec{e}}_{o}$ and ${\vec{e}}_{t}$ are calculated from the suture entry and exit points, whose values are obtained using the computer vision algorithm (33).

Using the aforementioned calculations, orthogonal and tangential forces for each suture location were obtained. For each active suturing time, (1)–(3) were used to compute metrics based on *F*_*o*_ and *F*_*t*_.

##### 2.1.4.3. Motion-based metrics

Metrics on total range of hand motion were extracted from IMU orientation data using (3), specifically PP(θ_*yaw*_), PP(θ_*pitch*_) and PP(θ_*roll*_) for each active suturing time (49).

##### 2.1.4.4. Physical contact metrics

The capacitive touch sensor was used to identify and count each instance of physical contact between the subject and the top and/or internal wall of the cylinder around the membrane holder. The total number of touches (*C*_*n*_) made during a suture cycle is used as a metric.

### 2.2. Experimental setup and protocol

Approval for the study was obtained from the applicable Institutional Review Board (Reference # Pro00011886). A total of 15 subjects (6 Attending Surgeons, 8 Surgery Residents and 1 Medical Student) were recruited from a local hospital to participate in the study. Informed consent was obtained from participants prior to participation. Each subject was asked to complete a questionnaire on their background and experiences. The data from 12 subjects (5 Attending Surgeons, 7 Surgery Residents) were used in analysis. The range of surgical suturing experience for attending surgeons was from 7 to 25 years, whereas the range of surgical suturing experience for residents was from 2 to 5 years. Three subjects did not meet the study criteria and were removed from analysis; 1 attending surgeon (did not meet subject pool definition, not actively practicing), 1 surgery resident (trial interruption), and 1 medical student (did not meet subject pool definition). All attendings in this study specialized in vascular surgery, except one who was a trauma surgery specialist.

Before suturing, subjects were encouraged to adjust the height of the table (Figure 1) to a comfortable level. The rationale for height adjustment was to allow users to choose a suitable height based on their individual physical characteristics and preferences. Participants were instructed to begin at 10 o'clock on the clock face and suture in a counter-clockwise fashion at each hour to complete a 12 hour cycle. At each hour, the needle is inserted at the marked location and withdrawn to make a stitch symmetric about the incision line. Subjects were instructed to perform continuous, uninterrupted suturing on the membrane using a Prolene suture needle (SH, 26 mm, 3-0) (Ethicon Inc., Somerville, NJ, USA). Subjects performed this procedure at two different membrane depths: at “surface” (i.e., 0 in. depth) and at “depth” (i.e., 4 in. depth) (Figure 2A).

Since the observed distribution of the metrics was not Gaussian (tested with Lilliefors test), the data were analyzed using the Wilcoxon rank sum test (5% significance level) to identify which metrics showed statistically different performance between attending and resident surgeons. Each stitch was considered as a separate trial. Suturing at the surface and at depth are analyzed separately.

## 3. Results and discussion

### 3.1. Metrics for skill assessments

**Figure 8:**
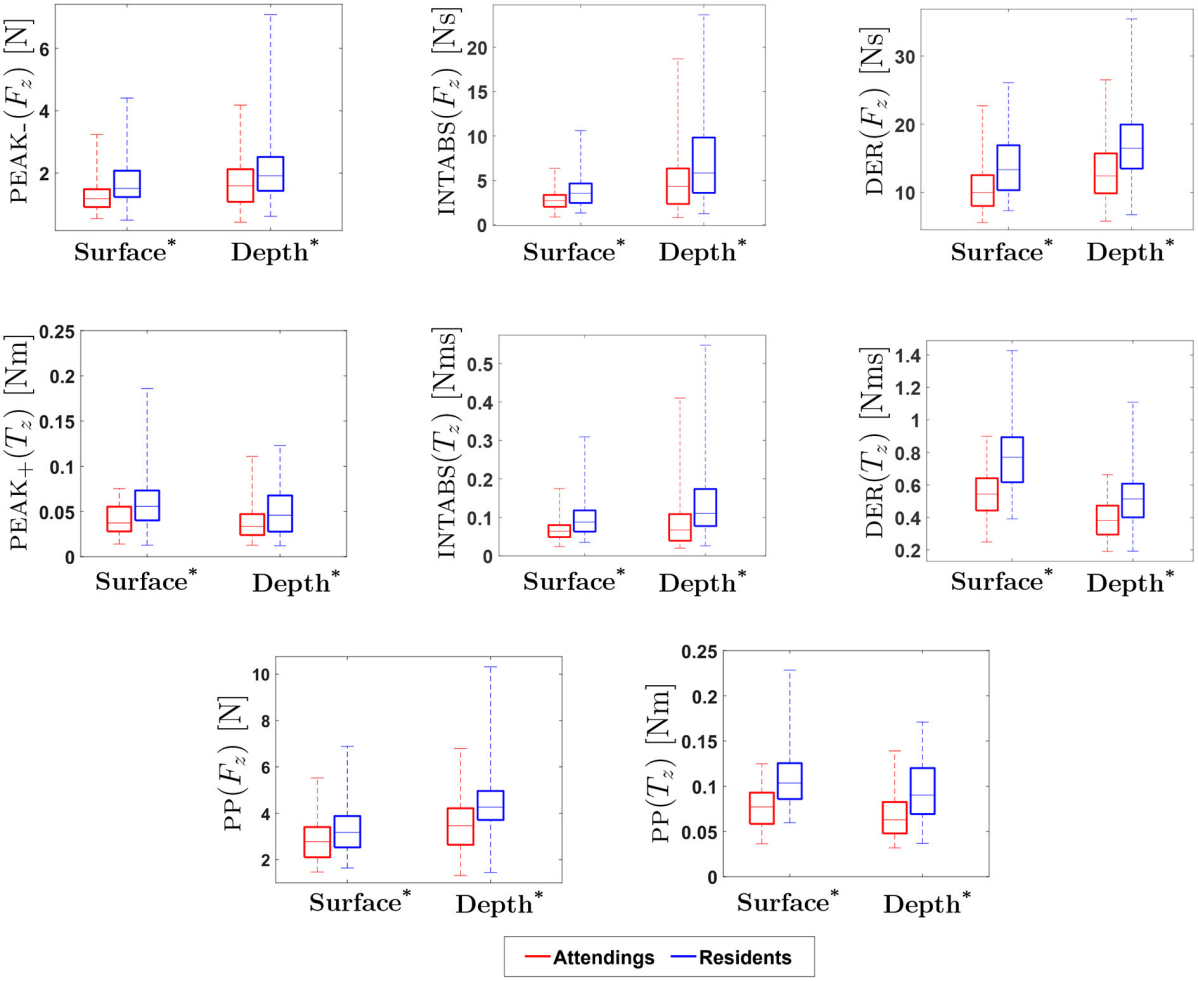
Experimental Results for Force/Torque-based Metrics: ^*^ indicates statistical significance for *p* < 0.05. (On each box, the middle line indicates the median, and the bottom and top edges of the box indicate the 25 and 75*th* percentiles, respectively. The whiskers are extended to the most extreme data points including outliers).

**Figure 9:**
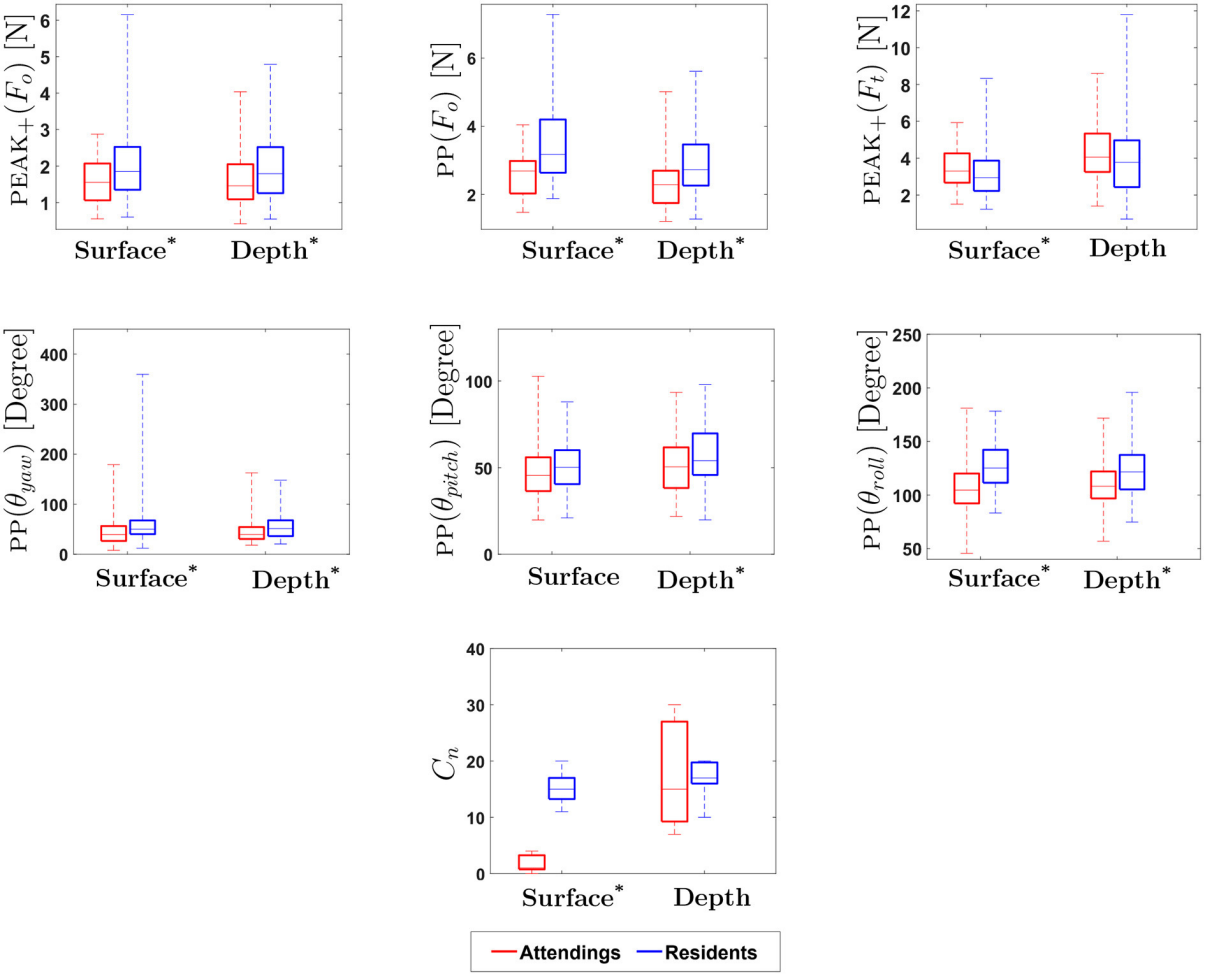
Experimental Results for Motion-based, Physical Contact and Vision-enabled Force Metrics: ^*^ indicates statistical significance for *p* < 0.05. (On each box, the middle line indicates the median, and the bottom and top edges of the box indicate the 25 and 75*th* percentiles, respectively. The whiskers are extended to the most extreme data points including outliers).

Tables 1, 2 show the *p*-values for statistical difference between attending and resident surgeons on various force, motion and touch metrics at surface level and at depth level. For selected metrics, Figures 8, 9 provide box plots of performance of attending and resident surgeons at surface level and at depth level. Interpretation and discussion of results are provided below.

**Table 1:** Statistical results for force/torque-based metrics.

		***p value***	

**Time series data**	**Metric**	**Surface**	**Depth**
	PEAK_+_(*F*_*x*_)	0.30	0.49
	PEAK_*-*_(*F*_*x*_)	0.18	0.75
*F*_*x*_(*t*)	PP(*F*_*x*_)	0.65	0.99
	INTABS(*F*_*x*_)	0.01^*^	0.25
	DER(*F*_*x*_)	<0.001^*^	0.51
	PEAK_+_(*F*_*y*_)	0.95	0.92
	PEAK_*-*_(*F*_*y*_)	0.14	0.24
*F*_*y*_(*t*)	PP(*F*_*y*_)	0.34	0.77
	INTABS(*F*_*y*_)	0.02^*^	0.06
	DER(*F*_*y*_)	<0.001^*^	0.34
	PEAK_+_(*F*_*z*_)	0.77	<0.001^*^
	PEAK_*-*_(*F*_*z*_)	<0.001^*^	0.01^*^
*F*_*z*_(*t*)	PP(*F*_*z*_)	0.01^*^	<0.001^*^
	INTABS(*F*_*z*_)	<0.001^*^	<0.001^*^
	DER(*F*_*z*_)	<0.001^*^	<0.001^*^
	PEAK_+_(*T*_*x*_)	0.54	0.17
	PEAK_*-*_(*T*_*x*_)	0.87	0.99
*T*_*x*_(*t*)	PP(*T*_*x*_)	0.39	0.43
	INTABS(*T*_*x*_)	0.04^*^	0.06
	DER(*T*_*x*_)	<0.001^*^	0.16
	PEAK_+_(*T*_*y*_)	0.29	0.40
	PEAK_*-*_(*T*_*y*_)	0.25	0.61
*T*_*y*_(*t*)	PP(*T*_*y*_)	0.12	0.84
	INTABS(*T*_*y*_)	0.01^*^	0.23
	DER(*T*_*y*_)	<0.001^*^	0.44
	PEAK_+_(*T*_*z*_)	<0.001^*^	0.01^*^
	PEAK_*-*_(*T*_*z*_)	<0.001^*^	<0.001^*^
*T*_*z*_(*t*)	PP(*T*_*z*_)	<0.001^*^	<0.001^*^
	INTABS(*T*_*z*_)	<0.001^*^	<0.001^*^
	DER(*T*_*z*_)	<0.001^*^	<0.001^*^

Metrics with statistical significance are shown with ^*^.

**Table 2:** Statistical results for motion-based, physical contact and vision-enabled force metrics.

		***p-value***	

**Time series data**	**Metric**	**Surface**	**Depth**
	PEAK_+_(*F*_*o*_)	<0.001^*^	0.02^*^
*F*_*o*_(*t*)	PEAK_*-*_(*F*_*o*_)	<0.001^*^	0.02^*^
	PP(*F*_*o*_)	<0.001^*^	<0.001^*^
	PEAK_+_(*F*_*t*_)	0.05^*^	0.12
*F*_*t*_(*t*)	PEAK_*-*_(*F*_*t*_)	0.02^*^	0.07
	PP(*F*_*t*_)	0.16	0.22
θ_*yaw*_(*t*)	PP(θ_*yaw*_)	<0.001^*^	<0.001^*^
θ_*pitch*_(*t*)	PP(θ_*pitch*_)	0.67	0.02^*^
θ_*roll*_(*t*)	PP(θ_*roll*_)	<0.001^*^	<0.001^*^
*C*(*t*)	*C*_*n*_	<0.001^*^	0.76

Metrics with statistical significance are shown with^*^.

#### 3.1.1. Force/torque-based metrics

Results for force-based metrics show that INTABS(*F*_*x*_), DER(*F*_*x*_), INTABS(*F*_*y*_) and DER(*F*_*y*_) were significantly different between attendings and residents at surface level. In addition, a statistical difference in performance between attending and resident surgeons was found for metrics PEAK_*-*_(*F*_*z*_), PP(*F*_*z*_), INTABS(*F*_*z*_) and DER(*F*_*z*_) at both depth and surface as well as for metric PEAK_+_(*F*_*z*_) at depth. For *z*-directional force metrics, the medians of attendings at both surface and depth level were found to be lower as compared to residents. Similar to an earlier study in laparoscopic suturing (17), our results show that *z*-directional force was found to be important for distinguishing between experience levels. In contrast to *z*-directional forces, in our study, metrics calculated for *x* and *y* direction forces at both surface and depth level were found to be non-significant.

Similarly, results for torque-based metrics show that INTABS(*T*_*x*_), DER(*T*_*x*_), INTABS(*T*_*y*_) and DER(*T*_*y*_) were significantly different between attendings and residents at surface level. In particular, results for torque-based metrics show that z directional torques (PEAK_+_(*T*_*z*_), PEAK_*-*_(*T*_*z*_), PP(*T*_*z*_), INTABS(*T*_*z*_) and DER(*T*_*z*_)) were significantly different between attendings and residents, at both the depth and surface level. The z-axis is vertical, so *T*_*z*_ is associated with forces orthogonal to the z-axis applied with a non-zero moment arm. Given the radial suturing pattern, that means *T*_*z*_ is most closely associated with forces orthogonal to the stitch direction. This motivates direct measurement of the orthogonal force *F*_*o*_, as explained in 2.1.4.2.

#### 3.1.2. Vision-enabled force metrics

Results show that the metrics obtained from orthogonal force (*F*_*o*_) were statistically different between attendings and residents on both surface and depth levels (see Table 2). In addition, tangential force (*F*_*t*_) metrics were significantly different between attendings and residents at surface, with the exceptions of PP(*F*_*t*_). Orthogonal forces applied by attendings were lower than those applied by residents, whereas tangential forces applied by attendings were higher.

In Horeman et al. (17), subjects made parallel sutures aligned with the *y* axis of the force sensor. It was observed that the maximum absolute forces in *x* and *y* directions were important for distinguishing between experience levels. Since the stitch direction was unchanged, *x* and *y* force directions were always orthogonal and tangential to the stitch direction, respectively. The study presented here uses a radial suture membrane with stitches in 12 different directions (see Figure 2B). This radial membrane is based on the one used in FVS training and is intended to test the subject's dexterity and preparedness for vascular anastomosis. Since the force sensor was fixed in place, *x* and *y* force directions were not generally aligned with stitch direction. Even though *x* and *y* directional force metrics were not found to be statistically significant in our study, measurements of forces in *x* and *y* directions are required to calculate orthogonal and tangential forces. Reinterpreting the *x* and *y* for axes from Horeman et al. (17) as orthogonal and tangential to stitch direction, the present study supports that orthogonal forces, and to a lesser extent tangential forces, are important for distinguishing skilled performance.

#### 3.1.3. Motion-based metrics

Previous studies suggest that there is a significant difference in hand movement between expert and novice surgeons during suturing. The rotation of the wrist, indicated by θ_*roll*_, was previously found to be particularly useful in assessment of suturing skill (17, 18). In the present study, similar to earlier studies, the total range of hand movement for PP(θ_*yaw*_) and PP(θ_*roll*_) at both surface and depth, and for PP(θ_*pitch*_) at depth were found to be statistically significant in differentiating attendings from residents. This suggests that yaw, pitch and roll might be useful for assessment of suturing skill.

Results for yaw, pitch, and roll show that total range of hand movement by attendings are consistently lower than that of residents, regardless of depth. In Dubrowski et al. (18) and Horeman et al. (17), it was found that experts use greater wrist rotation during suturing. In contrast, our results show that attendings use less wrist rotation. This may be explained by the fact that the majority of attendings in this study were experts in the field of vascular surgery. Due to the intricate nature of this type of surgery, it may be reasonable to assume that significant wrist rotation is not necessary in achieving accurate suturing during the surgical procedure. Also, pitch was found to be statistically significant, but only at depth, possibly because hand motion is more complicated when a subject sutures at depth. Moreover, during the experiments, it was observed that inexperienced participants tend to reposition the needle holder more often while suturing at depth. The complexity of hand movement during suturing deserves further investigation, specifically for suturing at depth, an essential aspect of vascular suturing.

#### 3.1.4. Physical contact metrics

We examined the number of times subjects made physical contact with the platform at both surface and depth conditions. Results indicate that the total number of physical touches (*C*_*n*_) on surface level for attendings was significantly lower than for residents, whereas there was no statistical difference between attendings and residents at depth. It should be noted that suturing at depth was introduced to mimic more realistic surgical conditions; however, feedback from attendings after the experiment revealed that requiring a surgeon to suture accurately without touching the top and/or the walls of the cylinder was an overly restrictive constraint. In fact, in certain conditions during surgery, surgeons strategically use boundaries of body cavities, for instance, to augment their forces during suturing.

## 4. Conclusion

In this paper, we presented a suturing simulator with the capability of collecting synchronized force, motion, touch, and video data to allow for the assessment of suturing skill in open surgery. Data collected from the simulator during suturing allowed for the extraction of metrics for quantifying suturing skill between different levels of trainees. Force-based, torque-based, motion-based, and physical contact metrics were presented. Combining force data with computer vision information, vision-enabled force metrics were found, specifically for forces orthogonal and tangential to stitch direction which provide deeper insight into suturing performance. Also, the vision algorithm aided in the identification of suture events and the segmentation of corresponding sensor data.

Experimental data collected from both attendings and residents were presented. Presented metrics were used to compare attendings' and residents' performance. Analysis shows that force metrics (force and torque in z direction), motion metrics (yaw, pitch, roll), physical contact metric, and image-enabled metrics (orthogonal and tangential forces) were statistically significant in differentiating suturing skill between attendings and residents. These results demonstrate the feasibility of distinguishing fine skill differences between attendings and residents, as compared to experienced vs. completely inexperienced personnel.

Limitations and Future Work: One key limitation of the current study is the small sample size. Consequently, while the results indicate the feasibility of the methods and metrics used, one cannot draw generalizable results regarding suture skill assessment from these results alone. Furthermore, the pool of attending surgeons in the study included mostly vascular surgeons. We are currently performing a large scale study of suturing skill assessment using the simulator with a wider range of surgical specialties. We hope that future work along these lines will enable the development of training methodologies to accelerate skill acquisition.

## Data availability statement

The datasets presented in this article are not readily available because of restrictions on data access placed by the relevant IRB. Requests to access the datasets should be directed to joseph@clemson.edu.

## Ethics statement

The studies involving human participants were reviewed and approved by Clemson University Institutional Review Board. Written informed consent for participation was not required for this study in accordance with the national legislation and the institutional requirements.

## Author contributions

IK contributed to the system design, data collection, data analysis and interpretation, and drafting and revising of the manuscript. JE contributed to the conception of the design and experiment design. RG contributed to the conception of the design, system design, data analysis and interpretation, and writing and revising the manuscript. RS contributed to the conception of the design, experiment design, data collection, and writing and revising the manuscript. All authors contributed to the article and approved the submitted version.

## Funding

This work was supported by the National Institutes of Health (NIH) Grant No. 5R01HL146843.

## Conflict of interest

The authors declare that the research was conducted in the absence of any commercial or financial relationships that could be construed as a potential conflict of interest.

## Publisher's note

All claims expressed in this article are solely those of the authors and do not necessarily represent those of their affiliated organizations, or those of the publisher, the editors and the reviewers. Any product that may be evaluated in this article, or claim that may be made by its manufacturer, is not guaranteed or endorsed by the publisher.
